# Association Between Obesity Type and Common Vascular and Metabolic Diseases: A Cross-Sectional Study

**DOI:** 10.3389/fendo.2019.00900

**Published:** 2020-01-09

**Authors:** Peng Zhang, Xin Sun, Hang Jin, Fu-Liang Zhang, Zhen-Ni Guo, Yi Yang

**Affiliations:** ^1^Clinical Trial and Research Center for Stroke, Department of Neurology, The First Hospital of Jilin University, Changchun, China; ^2^Department of Neurology, The First Hospital of Jilin University, Changchun, China

**Keywords:** obesity, waist circumference, metabolic diseases, stroke, coronary heart disease

## Abstract

**Background:** The association between different types of obesity and some chronic diseases in Dehui, Jilin province, China, is still unclear. The aim of our study was to clarify the association between different types of obesity and chronic diseases.

**Methods:** Residents aged 40 years or older were randomly selected using a multistage stratified cluster sampling method. Data were collected by means of face-to-face interview, physical examination, and laboratory examination. Descriptive data analyses were performed, and multiple logistic regression analyses were used to explore the adjusted association between different types of obesity and common vascular and metabolic diseases.

**Results:** The prevalence of general obesity alone, central obesity alone and compound obesity were 0.15, 54.29, and 14.36%, respectively. The prevalence of coronary heart disease, stroke, hypertension, dyslipidemia, and diabetes mellitus was highest in the compound obesity group, and lowest in the non-obesity group. Hypertension, dyslipidemia, and diabetes mellitus were associated with compound obesity and central obesity alone [compound obesity (OR = 4.703, 95% CI: 3.714–5.956 for hypertension; OR = 4.244, 95% CI: 3.357–5.365 for dyslipidemia; OR = 4.575, 95% CI: 3.194–6.552 for diabetes mellitus); central obesity alone (OR = 2.210, 95% CI: 1.901–2.570 for hypertension; OR = 2.598, 95% CI: 2.241–3.012 for dyslipidemia; OR = 2.519, 95% CI: 1.834–3.459 for diabetes mellitus)]. Coronary heart disease was associated with compound obesity (OR = 1.761, 95% CI: 1.141–2.719) but not central obesity alone (OR = 1.409, 95% CI: 0.986–2.013). Stroke was associated with neither compound obesity (OR = 1.222, 95% CI: 0.815–1.833) nor associated with central obesity alone (OR = 1.080, 95% CI: 0.786–1.485).

**Conclusions:** Central obesity alone and compound obesity are associated with the risk of hypertension, hyperlipidemia, and diabetes mellitus. Compound obesity but not central obesity alone is associated with the risk of coronary heart disease, but further research is needed to confirm it. There are no significant relationship between stroke and central obesity alone or compound obesity.

## Introduction

At the beginning of twentieth century, the main diseases threatening human health were acute and chronic infectious diseases. With improvements in health conditions, the popularization of vaccinations and the wide application of antibiotics, the incidence of infectious diseases has steadily decreased. In the latter half of the twentieth century, chronic diseases have gradually replaced infectious diseases as the forefront of Chinese disease concerns (1). The long duration, widespread prevalence, high cost, high mortality, and high disability rate of chronic diseases, if not effectively controlled in time, will bring a heavy burden to society, families, and individuals. The primary prevention of chronic disease is therefore essential to reducing disease burden.

The effects of abnormal body mass index (BMI) on chronic diseases are widely recognized (2, 3). Studies have also shown that hypertension, dyslipidemia, and diabetes were associated with abnormal waist circumference (WC) (4–6). However, central obesity, captured by using waist circumference, has been associated with atherosclerotic cardiovascular disease risk and may be missed when BMI is used as the only measure of obesity (7, 8). Therefore, it is recommended that all patients with BMI < 35 kg/m^2^ should measure waist circumference (9, 10). Nevertheless, not all diseases are associated with abnormal BMI or waist circumference. The INTERSTROKE study found that the risk of stroke was associated with abnormal waist-to-height ratio, but not with abnormal BMI or waist circumference (11). To sum up, BMI and waist circumference have different values for different diseases.

Obesity population can be divided into four categories according to BMI and waist circumference: non-obesity (with normal BMI and WC), general obesity alone (with normal WC and abnormal BMI), central obesity alone (with normal BMI and abnormal WC) and compound obesity (with abnormal BMI and WC). Different types of obesity may have different effects on chronic diseases. Exploring the relationship between different types of obesity and diseases can guide the rational selection of obesity indicators in the process of disease prevention. A newly published study has shown that central obesity without general obesity was found to be associated with an elevated risk of coronary heart disease while general obesity without central obesity did not (12). However, the association between different types of obesity and some chronic diseases in Dehui Jilin province, China, is still unclear. The purpose of our study was therefore to clarify the association between different types of obesity and chronic diseases, so as to guide the development of public health policy in Jilin province.

## Materials and Methods

### Study Design and Population

We conducted this population-based, cross-sectional study in Dehui, Jilin province, China. The detailed study protocol was described in a previous article (13). In summary, residents aged 40 years or older who lived in the area for more than 6 months were randomly selected using the multistage stratified cluster sampling method. The sample size (*n*) required for this cross-sectional study was calculated using a previously reported 2.37% prevalence rate (*p*) of stroke in adults aged 40 years or older in China (14), where the formula *N* = (${\text{Z}}_{\text{α}}^{2}$pq)/d^2^ (where Z_α_ = 1.96, α = 0.05, q = 1 – p, and d = 0.2p) was used. A total of 4,445 subjects were recruited for this study, of which 4,100 completed the survey, yielding a response rate of 92.23%. Forty-eight participants without electrocardiogram (ECG) or blood biochemical results were excluded. Finally, complete information from 4,052 participants was included in this study.

### Ethics Approval

The human ethics and research ethics committee of the First Hospital of Jilin University approved this study (approval No: 2015-R-250). All participants signed a written informed consent.

### Data Collection and Measurements

Data were collected by uniformly trained investigators by means of face-to-face interview and physical examination. Subject height was measured without shoes and weight was measured in light clothing. Waist circumference was measured according to the standard method (0.5–1.0 cm above the navel with the subjects breathing naturally). The subject's blood pressure was measured while sitting using an electronic sphygmomanometer (OMRON HEM-7200) after 20 min of rest, and the average value of two measurements was recorded. After fasting for at least 8 h, blood samples were collected in the morning and transported to the clinical laboratory through cold chain (Changchun Kingmed center for Clinical Laboratory Co., Ltd.). All blood samples were tested within 8 h of collection.

### Definitions

A stroke is defined by neurologists according to the criteria of the World Health Organization (WHO). Self-reported stroke patients were asked to provide their paper-based medical records. All participants underwent electrocardiogram (ECG) examination, and coronary heart disease (CHD) was defined according to the results of the ECG as read by physicians. Participants with TC ≥ 5.18 mmol/L, TG ≥ 1.70 mmol/L, HDL-C < 1.04 mmol/L, LDL-C ≥ 3.37 mmol/L or a previous diagnosis of hyperlipidemia by a physician were defined as having dyslipidemia (15). Diabetes mellitus was defined as a fasting plasma glucose (FPG) ≥ 7.0 mmol/L or a previous diagnosis of diabetes mellitus by a physician (16). Hypertension was defined according to the following criteria: systolic blood pressure ≥ 140 mmHg, diastolic blood pressure ≥ 90 mmHg and/or self-reported hypertension (17). Participants were divided into four categories for our study: non-obese, general obesity alone, central obesity alone, and compound obesity. BMI ≥ 28 kg/m^2^ were defined as abnormal BMI and WC ≥ 80 cm for a female or WC ≥ 85 cm for a male were defined as abnormal WC based on the Guidelines for Prevention and Control of Overweight and Obesity in Chinese Adults (18, 19). Participants with a normal WC but an abnormal BMI were defined as general obesity alone; participants with a normal BMI but an abnormal WC were defined as central obesity alone; participants with abnormal WC and BMI were defined as compound obesity; and participants with normal WC and BMI were defined as non-obese. Smoking was divided into three categories in our study: never smoking, former smoking, and current smoking. Those who were still consuming any type of tobacco products at the time of the survey were defined as current smokers. Former smokers were defined as people who had quit smoking for more than 3 months. People who never smoke were those who had never smoked or smoked <100 cigarettes in their lifetime (20). Drinking was defined as drinking more than 42 g of pure alcohol per day or 98 g of pure alcohol per week (21). The type of dietary pattern was determined by the participants themselves according to their own eating habits in the questionnaire. Exercise more than three times a week, each lasting more than 30 min, was defined as regular exercise.

### Statistical Analysis

Data were analyzed using the Statistical Program for Social Sciences, version 19.0 (IBM Corporation, Armonk, NY, USA). Data were present as absolute number and percentages. The chi-squared test was used to compare ratio differences between groups. The general obesity alone group accounted for 0.15% (6) of the total participants and had no significant effect on the main results, and was therefore deleted from subsequent statistical analysis. Multiple logistic regression analyses were used to explore adjusted association between different types of obesity and common vascular or metabolic diseases. Sensitivity analysis was conducted using two models. Age and sex were adjusted in model 1. Possible covariates were adjusted as much as possible in model 2 (different diseases adjusted different covariates). The aim of the sensitivity analysis was to get the upper and lower range of the OR value. The final results was based on model 2. All tests were two-tailed, and P < 0.05 was considered statistically significant.

## Results

A total of 4,052 participants with complete information were randomly selected from Dehui City, Jilin province, China. Their mean age was 54.85 ± 9.30. Male and urban subjects accounted for 40.0 and 51.0% of the total population, respectively. Detailed information were listed in Table 1. The proportions of general obesity alone, central obesity alone, and compound obesity in the study population were 0.15, 54.29, and 14.36%, respectively (Table 1).

**Table 1:** Characteristics associated with different types of obesity.

**Characteristics**	**Total**	**Non-obesity**	**General obesity alone**	**Central obesity alone**	**Compound obesity**
**Total**	4,052	1,264 (31.20)	6 (0.15)	2,200 (54.29)	582 (14.36)
**Sex**					
Male	1,619 (40.0)	531 (32.8)	5 (0.3)	812 (50.2)	271 (16.7)
Female	2,433 (60.0)	733 (30.2)	1 (0)	1,388 (57.0)	311 (12.8)
**Age (year)**					
40~	1,376 (34.0)	552 (40.1)	4 (0.3)	632 (45.9)	188 (13.7)
50~	1,372 (33.9)	407 (29.7)	2 (0.1)	754 (55.0)	209 (15.2)
60~	1,009 (24.9)	222 (22.0)	0 (0)	635 (62.9)	152 (15.1)
70~	295 (7.2)	83 (28.1)	0 (0)	179 (60.7)	33 (11.2)
**Area**					
Urban	2,067 (51.0)	693 (33.5)	4 (0.2)	1,090 (52.8)	280 (13.5)
Rural	1,985 (49.0)	571 (28.8)	2 (0.1)	1,110 (55.9)	302 (15.2)
**Education**					
Primary school and below	1,446 (35.6)	384 (26.6)	1 (0)	834 (57.7)	227 (15.7)
Junior middle school	1,696 (41.9)	524 (30.9)	5 (0.3)	928 (54.7)	239 (14.1)
Senior middle school	537 (13.3)	192 (35.7)	0 (0)	271 (50.5)	74 (13.8)
College and above	373 (9.2)	164 (44.0)	0 (0)	167 (44.7)	42 (11.3)
**Smoking**					
Current	1,373 (33.9)	449 (32.7)	3 (0.2)	730 (53.2)	191 (13.9)
Former	2,025 (50.0)	166 (25.4)	0 (0)	387 (59.2)	101 (15.4)
Never	654 (16.1)	649 (32.0)	3 (0.1)	1,083 (53.6)	290 (14.3)
**Drinking**					
Yes	1,074 (26.5)	337 (31.4)	4 (0.4)	556 (51.7)	177 (16.5)
No	2,978 (73.5)	927 (31.1)	2 (0.1)	1,644 (55.2)	405 (13.6)
**Regular exercise**					
Yes	3,150 (77.7)	993 (31.5)	5 (0.2)	1,720 (54.6)	432 (13.7)
No	902 (22.3)	271 (30.0)	1 (0.1)	480 (53.3)	150 (16.6)
**Dietary pattern**					
Balance	2,359 (58.2)	773 (32.8)	4 (0.2)	1,249 (52.9)	333 (14.1)
More meats	264 (6.5)	63 (23.8)	1 (0.4)	143 (54.2)	57 (21.6)
More vegetables	1,429 (35.3)	428 (30.0)	1 (0.1)	808 (56.5)	192 (13.4)

**Figure 1:**
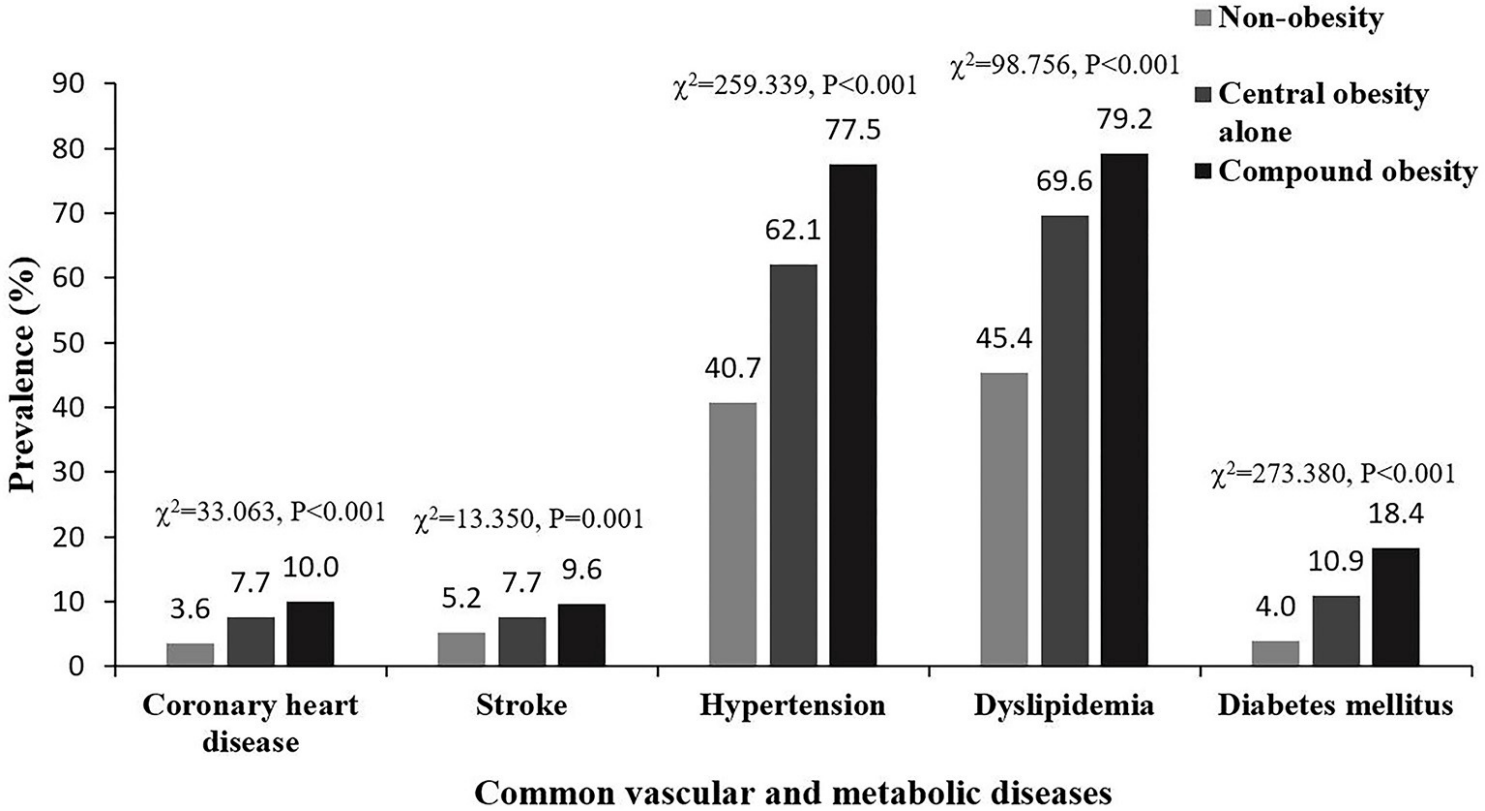
Prevalence of common vascular and metabolic diseases in different types of obesity.

Table 2 and Figure 1 show that the prevalence of coronary heart disease, stroke, hypertension, dyslipidemia, and diabetes mellitus was highest in the compound obesity group, and lowest in the non-obese group (compound obesity > central obesity alone > non-obese). The prevalence of these diseases increased with changes in obesity type, from non-obese to compound obesity. Chi-squared for trend values were 33.063 (coronary heart disease), 13.350 (stroke), 259.339 (hypertension), 98.756 (dyslipidemia), and 273.380 (diabetes mellitus), and the corresponding *p*-values for all comparison were <0.001.

**Table 2:** Chronic disease prevalence in patients with different types of obesity.

**Diseases**	**Non-obesity**	**Central obesity alone**	**Compound obesity**	**χ^**2**^**	***P***	***P* for trend**
**Coronary heart disease**				33.149	<0.001	<0.001
Yes	45 (3.6)	169 (7.7)	58 (10.0)			
No	1,219 (96.4)	2,031 (92.3)	524 (90.0)			
**Stroke**				13.399	0.001	0.001
Yes	66 (5.2)	170 (7.7)	56 (9.6)			
No	1,198 (94.8)	2,030 (92.3)	526 (90.4)			
**Hypertension**				258.366	<0.001	<0.001
Yes	515 (40.7)	1,363 (62.0)	451 (77.5)			
No	749 (59.3)	837 (38.0)	131 (22.5)			
**Dyslipidemia**				98.775	<0.001	<0.001
Yes	574 (45.4)	1,531 (69.6)	461 (79.2)			
No	690 (54.6)	669 (30.4)	121 (20.8)			
**Diabetes mellitus**				275.360	<0.001	<0.001
Yes	51 (4.0)	240 (10.9)	107 (18.4)			
No	1,213 (96.0)	1,960 (89.1)	475 (81.6)			

**Figure 2:**
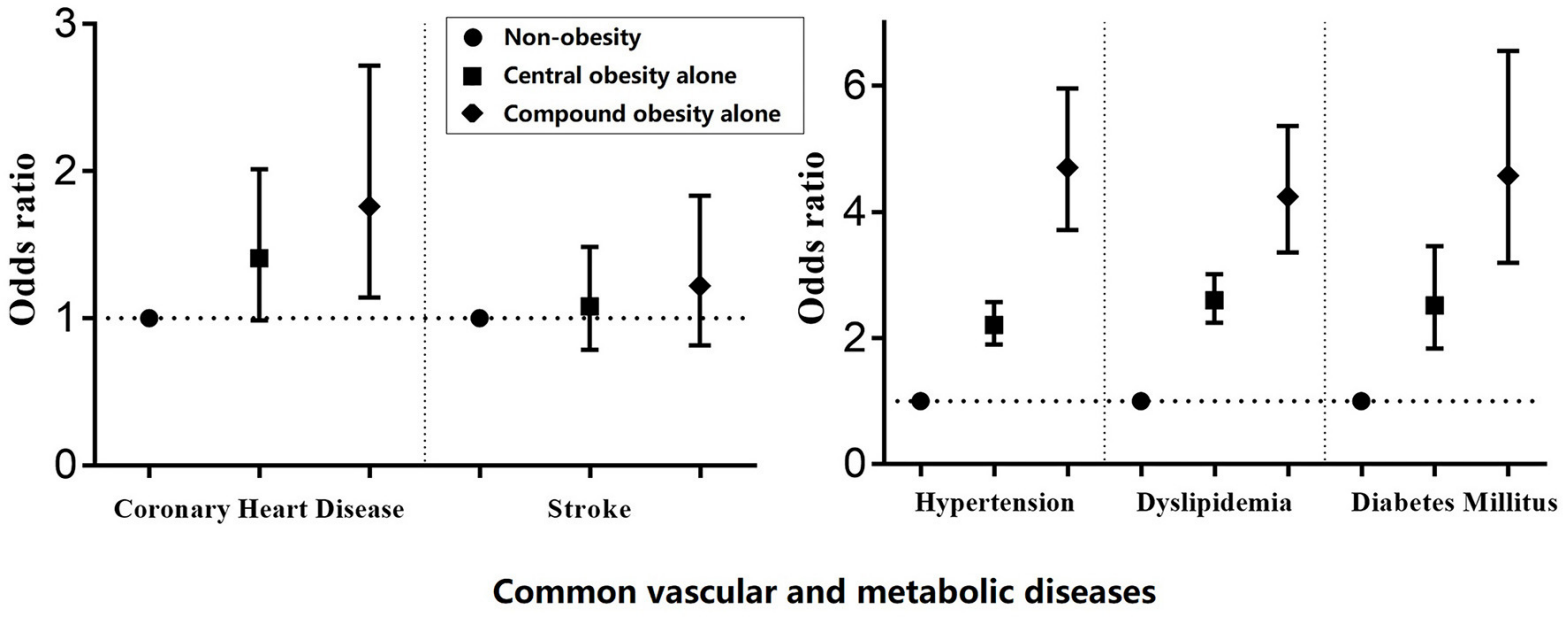
Adjusted association between common vascular and metabolic diseases and different types of obesity.

Table 3 and Figure 2 describe the association between diseases and different types of obesity. Hypertension, dyslipidemia, and diabetes mellitus were associated with compound obesity and central obesity only [compound obesity (OR = 4.703, 95% CI: 3.714–5.956 for hypertension; OR = 4.244, 95% CI: 3.357–5.365 for dyslipidemia; OR = 4.575, 95% CI: 3.194–6.552 for diabetes mellitus); central obesity only (OR = 2.210, 95% CI: 1.901–2.570 for hypertension; OR = 2.598, 95% CI: 2.241–3.012 for dyslipidemia; OR = 2.519, 95% CI: 1.834–3.459 for diabetes mellitus)]. Coronary heart disease was associated with compound obesity (OR = 1.761, 95% CI: 1.141–2.719).

**Table 3:** Associations between chronic disease and different types of obesity.

**Diseases**	**Non-obesity**	**Central obesity alone**	**Compound obesity**	***P* for trend**
**Coronary heart disease**				
Model 1^a^	Ref.	1.745 (1.238, 2.460)	2.618 (1.739, 3.942)	<0.001
Model 2^b^	Ref.	1.409 (0.986, 2.013)	1.761 (1.141, 2.719)	0.010
**Stroke**				
Model 1^a^	Ref.	1.296 (0.967, 1.756)	1.744 (1.191, 2.554)	0.004
Model 2^c^	Ref.	1.080 (0.786, 1.485)	1.222 (0.815, 1.833)	0.338
**Hypertension**				
Model 1^a^	Ref.	2.230 (1.926, 2.583)	4.859 (3.863, 6.112)	<0.001
Model 2^d^	Ref.	2.210 (1.901, 2.570)	4.703 (3.714, 5.956)	<0.001
**Dyslipidemia**				
Model 1^a^	Ref.	2.615 (2.262, 3.023)	4.435 (3.525, 5.581)	<0.001
Model 2^e^	Ref.	2.598 (2.241, 3.012)	4.244 (3.357, 5.365)	<0.001
**Diabetes mellitus**				
Model 1^a^	Ref.	2.606 (1.906, 3.566)	5.065 (3.561, 7.204)	<0.001
Model 2^f^	Ref.	2.519 (1.834, 3.459)	4.575 (3.194, 6.552)	<0.001

a *Adjusted for age and sex*.

b *Adjusted for age, sex, education, area, smoking, drinking, regular exercise, dietary pattern, hypertension, dyslipidemia, diabetes mellitus, and family history of coronary heart disease*.

c *Adjusted for age, sex, education, area, smoking, drinking, regular exercise, dietary pattern, hypertension, dyslipidemia, diabetes mellitus, and family history of stroke*.

d *Adjusted for age, sex, education, area, smoking, drinking, regular exercise, dietary pattern, and family history of hypertension*.

e *Adjusted for age, sex, education, area, smoking, drinking, regular exercise, dietary pattern, and family history of dyslipidemia*.

f *Adjusted for age, sex, education, area, smoking, drinking, regular exercise, dietary pattern, and family history of diabetes mellitus*.

## Discussion

Our study found that 68.8% of the residents of Dehui City, Jilin province were exposed to health hazards related to obesity (general obesity alone + central obesity alone + compound obesity), of which central obesity alone accounted for 54.29% of all residents. The prevalence of coronary heart disease, stroke, hypertension, dyslipidemia, and diabetes mellitus was highest in the compound obesity group, and lowest in the non-obese group. Moreover, the prevalence of these diseases in subjects with complex obesity was higher than in those with central obesity alone. Compound obesity and central obesity alone were both associated with the risk of hypertension, dyslipidemia, and diabetes mellitus. Coronary heart disease was associated with compound obesity but not central obesity alone, while stroke was associated with neither compound obesity nor central obesity alone.

Studies report that the prevalence of central obesity (central obesity alone + compound obesity in our study) varies from 39.0 to 57.4% in other regions of China (6, 22–28). This variability may be primarily attributed to lifestyle difference throughout China. Studies have shown that a variety of lifestyle factors can influence the incidence of central obesity, such as breakfast composition and sleep duration (29, 30). School education related to diet and lifestyle can reduce the incidence of central obesity in pre-teenagers, despite a tendency in this group toward decreased physical activity and increased screen time (31). In addition, different age limits for subject enrollment may have also contributed to the different rates of central obesity reported in these studies. Prior work indicates that the prevalence of central obesity is highest between 40 and 50 years old (32). Our study included only participants aged 40 years or older, which may have contributed to the higher prevalence of central obesity measured in this work. There are two commonly used definitions of central obesity based on waist circumference in China. One is that men with a waist circumference >90 cm and women with a waist circumference >80 cm are defined as centrally obese according to the International Diabetes Federation (33). However, whether this standard is applicable to Chinese population has not been confirmed. The other is that men with a waist circumference >85 cm and women with a waist circumference >80 cm are defined as centrally obese based on the Guidelines for Prevention and Control of Overweight and Obesity in Chinese Adults (19). The definition of central obesity in this guideline was based on the findings of the Working Group on Obesity in China (WGOC). After 2000, in order to establish the suitable diagnostic standard of overweight and obesity for Chinese adults, the Working Group on Obesity in China under the support of International Life Science Institute Focal point in China, whose members include experts and scholars in the fields of epidemiology, clinical medicine (cardiovascular, endocrine), etc. The working group has collected 13 large-scale population studies in China, involving more than 110,000 subjects from 21 provinces, cities and autonomous regions in China. Based on the results of the study, the working group proposed the cut point of waist circumference (≥85/80 cm, male/female) for the diagnosis of central obesity in Chinese adults (18). This standard has been confirmed to be suitable for Chinese people by several subsequent studies (34, 35). Therefore, the latter definition was adopted in our study. This may also explain why a higher prevalence of central obesity was measured in our study.

Our study showed that both compound obesity and central obesity alone were associated with hypertension, dyslipidemia, and diabetes mellitus, a finding that suggested that measuring resident waist circumference and BMI may prevent obesity-related diseases. Although there are different definitions of obesity based on BMI, an abnormal BMI has been shown to be an independent risk factor for hypertension, dyslipidemia, and diabetes mellitus (36–38). At present, the most commonly used way to prevent obesity-related diseases is to control excessive BMI. Although an abnormal BMI was associated with hypertension, dyslipidemia, and diabetes mellitus in this work, our study implied that focusing only on abnormal BMI values still puts a large proportion of the population at risk of chronic disease. People with an abnormal waist circumference and a normal BMI are easily neglected in the primary prevention of chronic disease (39). The health department of Jilin province should therefore take measures to improve people's awareness of waist circumference and central obesity in order to reduce the prevalence of hypertension, hyperlipidemia, and diabetes mellitus. Coronary heart disease was associated with compound obesity [OR = 1.761 (95% CI: 1.141–2.719)] but not central obesity alone in our study [OR = 1.409 (95% CI: 0.986–2.013)]. Although the relationship between coronary heart disease and central obesity alone was found to be negative, the OR value was large and the lower limit of the OR value was close to 1. We therefore could not conclude that an abnormal waist circumference had no harmful effect on coronary heart disease, as this negative relationship may have been caused by the excessive adjustment of covariates. Waist circumference therefore may still be significant in the prevention of coronary heart disease. Strokes were associated with neither compound obesity nor central obesity alone in our study, which means that controlling an abnormal waist circumference or BMI will not reduce the prevalence of strokes. Other indicators for defining obesity need to be found to better prevent strokes.

The strengths of our study lie in its representative sampling survey and high response rate. However, there are still some limitations. First, the cross-sectional design of this study limited its ability to reveal a causal link between chronic disease and a different type of obesity. Second, self-reported questionnaire interviews may be a potential source of bias. Third, there were not enough people with general obesity alone in our study, which made it impossible to explore the relationship between general obesity alone and chronic diseases.

## Conclusions

A large proportion of the residents of Dehui City, Jilin province are obese. Central obesity alone and compound obesity are associated with the risk of hypertension, hyperlipidemia, and diabetes mellitus. The relationship between waist circumference and these diseases should not be ignored. Compound obesity but not central obesity alone is associated with the risk of coronary heart disease, but further research is needed to confirm it. There are no significant relationship between stroke and central obesity alone or compound obesity.

## Data Availability Statement

All datasets generated for this study are included in the article/supplementary material.

## Ethics Statement

The studies involving human participants were reviewed and approved by The human ethics and research ethics committee of the First Hospital of Jilin University. The patients/participants provided their written informed consent to participate in this study.

## Author Contributions

YY and XS: conception and design. F-LZ and HJ: acquisition of the data. PZ: data analysis and drafting the manuscript. Z-NG and YY: critical revision. All authors: approved the final version for publication.

### Conflict of Interest

The authors declare that the research was conducted in the absence of any commercial or financial relationships that could be construed as a potential conflict of interest.
